# Serological and Virological Evidence of Crimean-Congo Haemorrhagic Fever Virus Circulation in the Human Population of Borno State, Northeastern Nigeria

**DOI:** 10.1371/journal.pntd.0005126

**Published:** 2016-12-07

**Authors:** David N. Bukbuk, Stuart D. Dowall, Kuiama Lewandowski, Andrew Bosworth, Saka S. Baba, Anitha Varghese, Robert J. Watson, Andrew Bell, Barry Atkinson, Roger Hewson

**Affiliations:** 1 Department of Microbiology, Faculty of Science, University of Maiduguri, Maiduguri, Borno State, Nigeria; 2 National Infection Service, Public Health England, Salisbury, Wiltshire, United Kingdom; 3 Animal Virus Research Laboratory, Department of Veterinary Microbiology and Parasitology, University of Maiduguri, Maiduguri, Borno State, Nigeria; University of Thessaloniki, Greece, UNITED STATES

## Abstract

**Background:**

Despite several studies on the seroprevalence of antibodies against Crimean-Congo Haemorrhagic Fever virus (CCHFV) from humans and cattle in Nigeria, detailed investigation looking at IgG and IgM have not been reported. Additionally, there have been no confirmed cases of human CCHFV infection reported from Nigeria.

**Principal Findings:**

Samples from sera (n = 1189) collected from four Local Government Areas in Borno State (Askira/Uba, Damboa, Jere and Maiduguri) were assessed for the presence of IgG and IgM antibodies. The positivity rates for IgG and IgM were 10.6% and 3.5%, respectively. Additionally, sera from undiagnosed febrile patients (n = 380) were assessed by RT-PCR assay for the presence of CCHFV RNA. One positive sample was characterised by further by next generation sequencing (NGS) resulting in complete S, M and L segment sequences.

**Conclusions:**

This article provides evidence for the continued exposure of the human population of Nigeria to CCHFV. The genomic analysis provides the first published evidence of a human case of CCHFV in Nigeria and its phylogenetic context.

## Author Summary

Crimean-Congo haemorrhagic fever (CCHF) is an acute tick-borne zoonotic disease. The causative agent, CCHF virus (CCHFV), has the most extensive geographical distribution of the medically important tick-borne viral diseases with a distribution over much of Asia, the Middle East, Africa and expanding areas of south-eastern Europe. Whilst the main route of human infection with CCHFV is predominantly through tick bite, it can also be spread via bodily fluids and it has a reputation for causing nosocomial outbreaks in healthcare settings. Whilst CCHFV has been identified in ticks from Nigeria since 1970, there is scarce information on human infection. Within this report, the prevalence of CCHFV-reactive antibodies has been assessed in human sera providing evidence of continued circulation of the virus in the human population of Borno state, Nigeria. Additionally, in one sample the presence of viral RNA was detected which allowed a full sequence of the CCHFV to be obtained. This is the first report of CCHFV being associated in a human case from Nigeria and the full genetic characterisation of the virus being completed. The evidence within supports the hypothesis that CCHFV is endemic in Nigeria and should be considered as an aetiological agent in febrile patients.

## Introduction

Crimean-Congo haemorrhagic fever (CCHF) is a disease caused by the CCHF virus (CCHFV) a member of the *Nairovirus* genus, family *Bunyaviridae* [1]. The disease was first reported in the Crimean peninsula in the mid-1940s, after a large outbreak of severe haemorrhagic fever, described as Crimean haemorrhagic fever (CHF), occurred with a case fatality rate of >30% [2]. The virus aetiology of CHF was not determined until the late 1960s, when it was subsequently shown to be antigenically identical to the Congo virus identified in Africa in 1967 [3,4]. The naming of the virus went through a series of steps before the name Crimean-Congo haemorrhagic fever virus was agreed in 1979 [5]. The disease is now endemic in many countries in Europe, Asia and Africa [1]. In nature, CCHFV is maintained predominantly in *Ixodid* tick vectors, particularly ticks of the genus *Hyalomma* [6]. Whilst tick bite is the most common route of CCHFV infection, person-to-person transmission can occur via direct exposure to blood or other secretions [6]. Direct zoonotic transmission from viremic animal hosts is also possible [7].

The first report of CCHFV in Nigeria occurred in 1970, when it was identified in various tick species, including *Hyalomma spp*. collected from market animals, and hedgehogs [8]. Interestingly, very few cases of CCHF have been recorded in Africa [9]; the majority are described from South Africa [10]. The risk of CCHF in several African countries is poorly defined and infection with CCHFV is often undiagnosed or unreported in these regions [11]. Importantly, CCHFV is a notorious cause of nosocomial infections especially when undiagnosed, and the virus presents a significant risk to health care workers [12–16].

A previous study of CCHFV conducted within Borno State, Nigeria reported a seroprevalence of 2.4% (7 out of 297 individuals) where the reservoir, vectors and intermediate hosts abound in the area [3]. In the present study, seroprevalance was expanded across different Local Government Areas (LGAs) of Borno State and samples from patients with undiagnosed febrile illness were assessed for the presence of CCHFV RNA.

## Methods

### Ethics statement

Blood samples tested in this study were previously taken for the laboratory diagnosis of malaria, typhoid or hepatitis and were classified as clinical specimens. No samples were collected specifically for this work, thus ethical approval for the study design was not required. Samples were anonymised within Nigeria, so investigators were only supplied with sequentially numbered samples. For the sample that was identified as positive for CCHFV RNA, the local team in Nigeria were able to provide more information on the background of the case. The patient described here is anonymous. Blood samples were collected and stored within the University of Maiduguri Teaching Hospital, Nigeria. Samples for testing at Public Health England, UK were sent in accordance with national guidelines for both Nigeria and the UK.

### Samples

**Fig 1:**
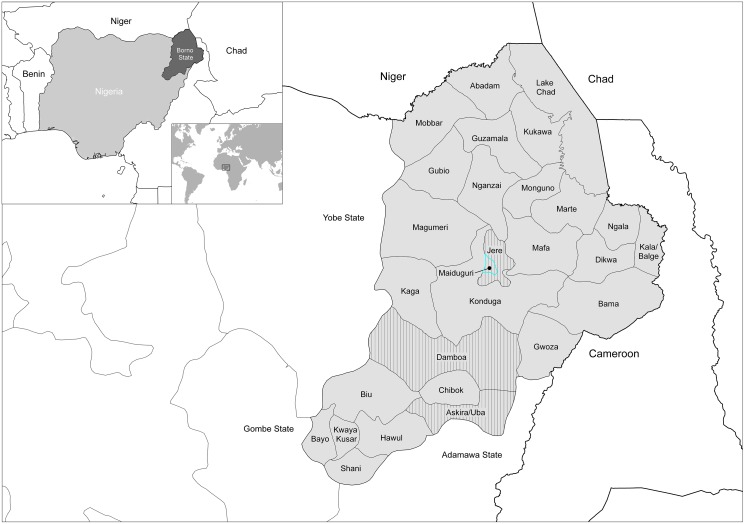
Map of Borno State showing the 27 Local Government Areas (the vertical fill denotes the 4 study areas).

Samples for serology testing were randomly selected from a cross section of humans in both rural and urban populations. Patients generally presented with febrile illnesses, and were screened for common aetiological agents, such as malaria and typhoid. They were associated with different occupational groupings which included abattoir workers, students, civil servants, and unemployed people, and they were of different age groups and genders. They presented to different clinics during 2010–2014. Samples were collected from 4 out of 27 LGAs within Borno State, namely: Askira/Uba, Damboa, Jere, and Maiduguri (Fig 1). Samples were heat treated at 56°C for 30 minutes prior to testing. The nature of the sampling sites is listed in Table 1. Within Askira/Uba is the town of Lassa where the first case of Lassa fever was reported in 1969 [17]. An abattoir within Maiduguri city, Maiduguri Metropolitan Abattoir (MMA), serves as the major animal slaughterhouse for the region. Animals (camel, cattle, goat and sheep) are brought from within the State (Borno), from neighbouring states and also from neighbouring countries such as Cameroon, Chad, Niger, Sudan and Central Africa.

**Table 1:** Number of samples tested from the Local Government Areas (LGA) of Borno State, Nigeria.

S/No.	Sample Notation	LGA (Domicile)	No. samples tested
1.	Askira/Uba	Askira/Uba (Rural)	98
2.	Dambua	Damboa (Rural)	235
3.	Synthyche	Jere (Urban)	77
4.	MJ	Jere (Urban)	369
5.	Vivian	Jere (Urban)	81
6.	Queen	Maiduguri (Urban)	251
7.	Abattoir	Maiduguri (Urban)	78
	Total		1,189

For molecular analysis, 380 serum samples from febrile patients in the acute phase of illness (fever and/or headache) were tested. These samples were from patients had previously been tested and found negative for both malaria and typhoid.

### Serology testing

Initial serology testing was conducted in Nigeria using an in-house ELISA to detect IgG and IgM antibodies against recombinant CCHFV nucleoprotein [18]. A selection of samples was shipped to the UK for confirmatory analysis using both the in-house ELISA and a commercially-available assay system (Vector-Best, Russia). For the latter, the manufacturer’s instructions supplied with the kits were followed.

### Molecular testing

Extraction of RNA was performed using the MagnaPure 96 small volume RNA kit (Roche). Plates were loaded onto the MagnaPure 96 automated extraction robot and RNA was eluted in 60μl nuclease free water. Target amplification was performed using primers to the CCHFV S segment [19] with a Superscript^®^ Platinum One-Step III qRT-PCR Kit (Life Technologies). Amplification was performed using the ABi 7500 (Applied Biosystems) at the following cycling conditions: 50°C for 10 minutes, 95°C for 2 minutes followed by 40 cycles of 95°C for 10 seconds and 60°C for 40 seconds; and a cooling cycle of 40°C for 30 seconds. Temperature cycling was set to maximum ramp speed and data were acquired and analysed using the ABi 7500 on-board software with an automatically selected threshold.

### Whole genome sequencing

The isolated RNA was treated with DNAse I (Life Technologies) following manufacturer’s instructions. After DNAse inactivation, the sample was cleaned up using a Zymo Clean and Concentrator column and eluted into 6 μl H_2_O.

A 5μl aliquot of the DNase I-treated RNA was used to prepare cDNA using the Ovation^®^ RNA-Seq System V2 (NuGen) following manufacturer’s instructions, with the exception that RNA was denatured for 5 min at 85°C prior to first strand synthesis. cDNA was purified using a MinElute Reaction Cleanup Kit (Qiagen) and eluted in 15 μl H_2_O. Final cDNA concentration was determined using the QuBit broad-range double-stranded DNA assay (Life Technologies).

An Illumina sequencing library was prepared using the Nextera XT V2 kit with 1.5 ng of cDNA as input, following manufacturer’s instructions. Indices were selected using the Illumina experiment manager software. Fragment size analysis was performed using a bioanalyser (Agilent Technologies). The KAPA library quantification kit for NGS (Kapa Biosystems) was used for quantification. The prepared sequencing library was run on an Iluminia MiSeq by the PHE Genomics Services Unit (GSDU).

### Bioinformatics analysis

Reads were trimmed to remove adaptors and low quality bases, to achieve an average phred score of Q30 across the read, using trimmomatic [20]. Trimmed reads were taxonomically assigned using Kraken [21] (ver.0.10.4-beta) populated with bacterial, viral and archaeal genomes and a representative yeast genome, from RefSeq (ver. 66) with the addition of 141 viral GenBank sequences.

Following Kraken assignment, viral reads were extracted from the fastq files using seq_select_by_id [22], and assembled using SPADES (ver. 3.1.1) [23]. All reads were then used to scaffold these contigs using SSPACE (ver. 1.0.5) [24]. Contigs larger than 1 kb were aligned to the CCHFV reference sequence (NC_005301.3, NC_005300.2 and NC_005302.1 for L, M and S segments, respectively), using Mauve Contig Mover (ver. 1.0.0) [25].

Reads were mapped to both assembled contigs and the CCHFV reference (NC_005301.3, NC_005300.2 and NC_005302.1) using BWA (ver. 0.7.5) [26]. Consensus genome sequence was produced at a minimum depth of five reads using an in-house script. All of the above was performed using a local instance of the Galaxy Project [27–29]. BAM files were visualised using tablet [30].

### Phylogenetic analysis

Phylogenetic analyses were performed using MEGA 6 [31]. Trees were precomputed using the Neighbour-Joining method [32], then evolutionary history and distances were inferred by the Maximum Likelihood method. Maximum Likelihood phylogenetic trees were generated for the open reading frames of the partial L, M and S sequences recovered from next-generation sequencing. All positions containing gaps and missing data were eliminated from the analysis.

## Results

### Serology testing

Of the 1,189 sera from the 4 LGAs tested, 126 were positive for CCHFV IgG giving an overall seroprevalence of 10.6%, while 42 (3.5%) and 7 (0.6%) were seropositive for IgM or IgG+IgM antibodies, respectively (Table 2). The study shows that the prevalence of IgG was higher in rural (15%) than in urban (8.9%) areas; conversely the incidence of IgM was slightly higher in urban (3.9%) than in rural (2.7%) areas.

**Table 2:** Serosurveillance results of CCHFV-specific antibody responses in populations from different areas in Borno State, Nigeria.

LGA (Domicile)	No. samples tested	No. (%)IgG positive	No. (%)IgM positive	No. (%)IgG+IgM positive
Askira/Uba (Rural)	98	23 (23.5)	4 (4.1)	0 (0.0)
Damboa (Rural)	235	27 (11.5)	5 (2.1)	1 (0.4)
Jere (Urban)	527	38 (7.2)	28 (5.3)	5 (0.9)
Maiduguri (Urban)	329	38 (11.6)	5 (1.5)	1 (0.3)
Total	1,189	126 (10.6)	42 (3.5)	7 (0.6)

### Molecular testing

Of 380 samples assessed for the presence of CCHF viral RNA by RT-PCR, a single sample (ID: N428) was positive with a cycle threshold (CT) value of 29.42 and a slope formation typically seen for a positive sample. The RNA from this sample was used for further characterisation.

### Whole genome sequencing results

Kraken taxanomic analysis placed 0.43% of reads as the species CCHFV. For confirmation, these reads were assembled into 136 contigs larger than 200bp. A nucleotide BLAST of these contigs agreed with the Kraken taxonomic analysis in identifying 12 contigs with typically greater than 95% identity with CCHF Sudan ABI_2009. Using Mauve contig mover the 136 contigs were aligned to the CCHFV reference genome where it was found that an assembled 8 contigs aligned to 93.26% of the viral genome. In order to assess the support for these assembled contigs, reads were mapped back to contigs and gave an average coverage of 61.39, 73.38, 34.71, 21.12, 16.38, 22.85, 198.4 and 115.17 fold across each contig respectively.

**Fig 2:**
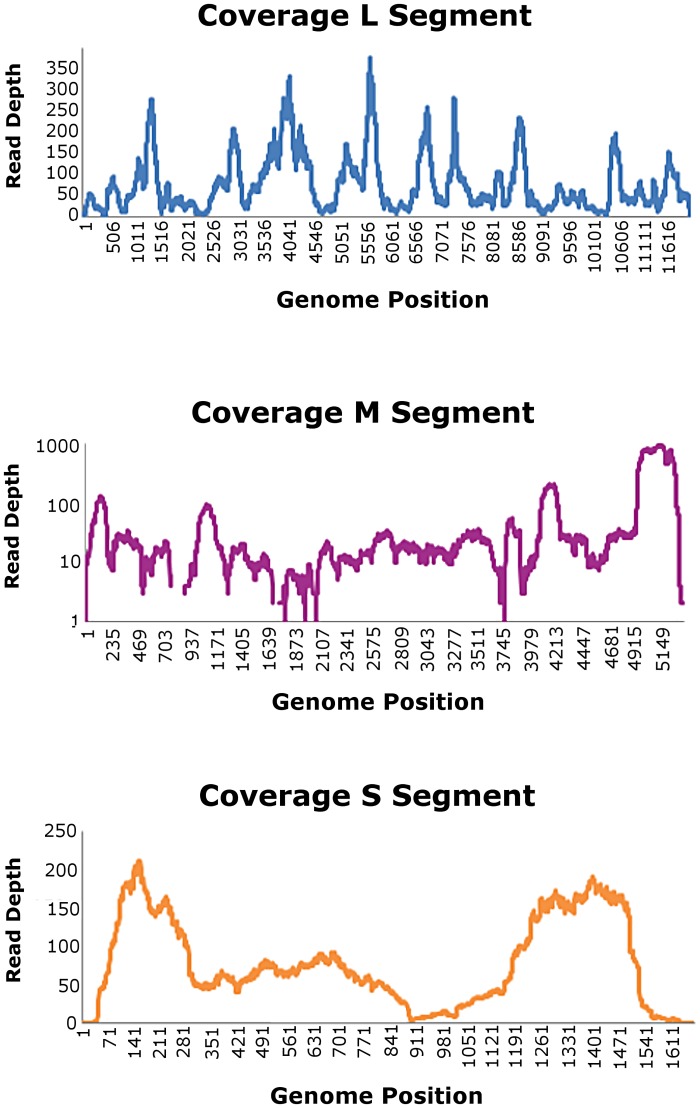
Coverage graphs of reads mapped to the three segments of the CCHF reference genome.

In parallel, reads were mapped to the CCHFV reference genome (NC_005301.3, NC_005300.2 and NC_005302.1) which gave 94.54% coverage of the full genome at a minimum depth of 5 bases (99.47% coverage for the L segment, 89.73% for the M segment and 94.43% for the S segment) and an average depth of coverage across the genome of 73.3 fold (with an average depth of 66.2 reads for the L segment, 71.2 for the M segment and 82.5 for the S segment). A consensus genome sequence was produced at a minimum depth of five reads. Sequences were submitted to GenBank with accession numbers KX238956-KX238958. Near full genome coverage was seen for the sample when mapped to CCHFV (Table 3). These mapping data are illustrated in Fig 2.

**Table 3:** Coverage of reads from next-generation sequencing to the segments of CCHF virus.

Segment	Mapped reads	Minimum coverage	Maximum coverage	Average coverage	Average length (zero coverage regions)	Fraction of reference covered
S	1279	0	225	82.539	31	0.963
M	3690	0	970	71.247	56.2	0.948
L	7990	0	333	66.205	8	0.999

**Fig 3:**
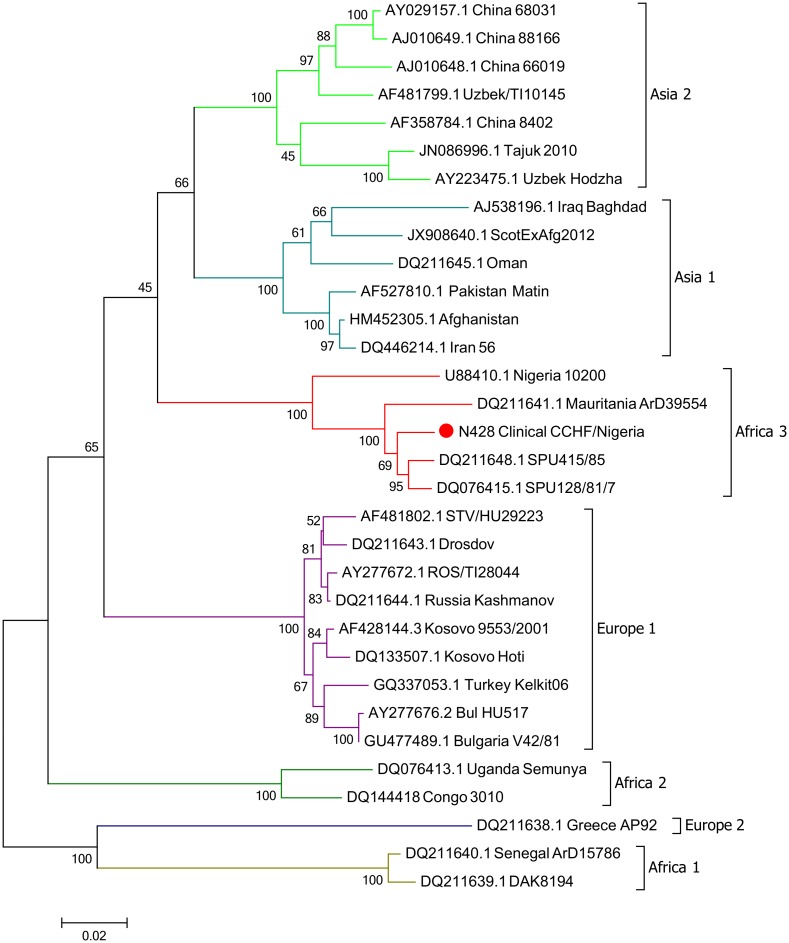
Maximum-Likelihood phylogenetic tree showing relationship distances obtained by comparing CCHFV S-segment open-reading frames. Different CCHFV clades are indicated by coloured lines.

**Fig 4:**
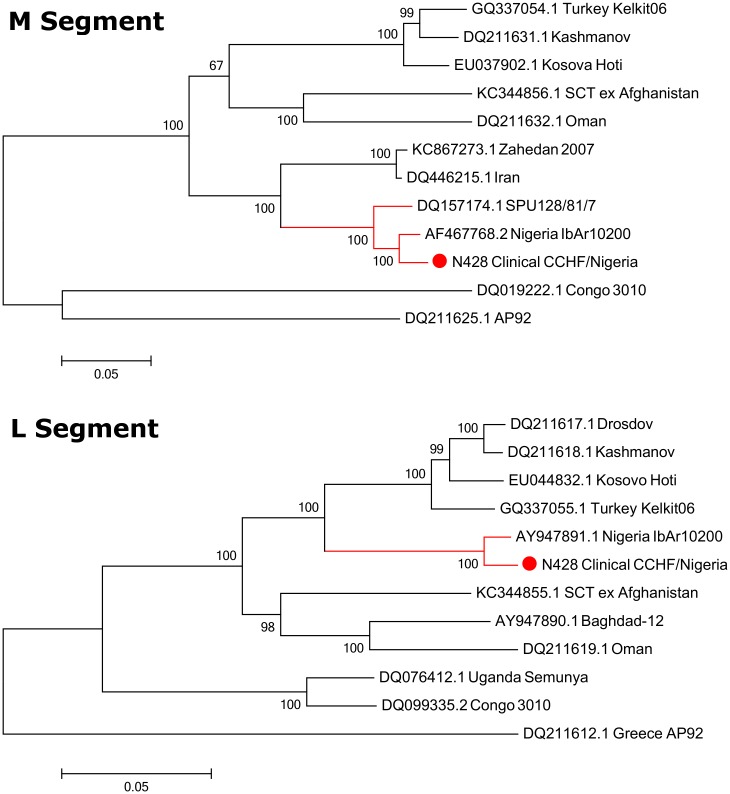
Maximum-Likelihood phylogenetic tree showing relationship distances obtained by comparing CCHFV M- and L-segment open-reading frames.

The genome assembly included three segments; the L Segment [GenBank KX238956], M Segment [GenBank KX238957] and S Segment [GenBank KX238958]. Phylogeny was inferred using the maximum likelihood method based on the Tamura-Nei model [33] and confidence assessed with the Bootstrap Test with 1000 resamplings. Phylogenetic analysis clustered the S segment in the Africa 3 phylogenetic group (Fig 3). The S segment open-reading frame showed close homology with a previous isolate of CCHFV from Nigeria (IbAr10200), as well as isolates from Mauritania (ArD39554) and South Africa (SPU415/85 and SPU128/61/7). The M and the L clustered closely with the Sudan AB1-2009 isolate and the Nigeria IbAr10200 isolate (Fig 4).

## Discussion

Our serological results demonstrate the circulation of CCHFV in different LGAs of Borno State, Nigeria. The average positivity rates were 10.6% for IgG responses and 3.5% for IgM responses. A previous study in Borno State using 297 samples collected from patients attending health facilities between September 2011 and February 2012 showed a prevalence rate for IgG antibodies against CCHFV of 2.4% using a similar ELISA test to that used in this study [34]. The difference in IgG levels may be due to different study populations, for example our report was conducted on samples collected from 4 LGAs whereas the earlier report was conducted on samples from 10 LGAs [34]. In 1974, it was reported that 9.6% of 250 sera collected in Nigeria had neutralisation activity specific to CCHFV [35]. As neutralisation activity was not performed in our study, cross-reactivity with other nairoviruses might be considered. The similarity in antibody responses between our study and that conducted in 1974 indicates that despite the 38 year time period, similar frequencies of human CCHFV infection remain in Nigeria. This is consistent with recent evidence consensus of a moderate level of CCHFV in Nigeria [22]. Given the expanding population of working Nigerians [36], there may be a greater likelihood of tick / CCHFV exposure in the future.

Although the data are focused on Borno State, we speculate that CCHFV is circulating in neighbouring countries which share common borders (Cameroon, Chad and Niger). Furthermore these borders are porous, and unrestricted human and animal movements are common throughout the year. Since the natural lifecycle of the *Hyalomma* tick involves feeding on cattle, CCHFV infectivity of cattle can help support data on human cases. In northern Nigeria, around 25.7% of sera collected from cattle showed antibody responses to CCHFV as tested by agar diffusion precipitation tests [37].

For molecular testing, sera samples from undiagnosed febrile patients were assessed for the presence of CCHFV using RT-PCR. Of the 380 samples tested, only a single sample showed a positive signal. This sample (N428) was obtained from a 15 year old female patient who was resident in old Maiduguri, a settlement almost at the outskirt of Maiduguri city. She was admitted in March 2012 into the female medical ward of the University of Maiduguri Teaching Hospital, a tertiary health facility in northeastern Nigeria with a 6 day history of fever, body pain, bloody diarrhoea and epistaxis. NGS of RNA from sample N428 identified the presence of CCHFV. Phylogenetic characterisation of the viral S segment sequence demonstrated that it belonged to the Africa 3 clade, in congruity with reports for other viruses isolated from Nigeria and similar geographies [6,19]. The patient went on to make a full recovery.

Unfortunately, since samples were heat-treated, virus isolation could not be performed. This is a common issue, exacerbated by difficulties and delays in transportation to appropriate containment facilities [38]. Previously only partial genomic sequence has been attainable in such cases [38,39]. However, the advent and use of NGS technologies here has enabled the efficient and rapid, characterisation of a clinical strain of CCHFV from Nigeria. Analysis of the genetic relationship between this CCHFV and previously characterised isolates shows close homology to the IbAr10200 strain, isolated from *Hyalomma excavatum* ticks in Sokoto, Nigeria in 1966 [40]. In addition to being a tick virus, this strain has also been passaged multiple times in the laboratory. It is interesting that despite being isolated 50 years ago from a tick, there is remarkable similarity in these Nigerian viruses. Analogous observations have been made in the past [41,42], illustrating that CCHFV can be genetically very stable over long periods of time, while on other occasions there is vast genetic variation between strains, even between those sequenced from similar locations [43]. Such observations may point to the broader ecological conditions which support the virus-host environment; highly changeable ecologies resulting in more opportunities for CCHF viruses to exploit new sequence space, whereas stable ecological conditions would restrict diversity. Whilst there has been strong serological evidence for CCHFV circulation in humans and cattle in Nigeria in the past, our data are the first to detect and directly sequence viral RNA in a human sample. Thus, this is the first report of human CCHFV infection in Nigeria. Importantly our work highlights that CCHFV should be considered as a potential cause of febrile illness in patients within the region and that further studies on the risk of CCHFV infection and how such risks could be reduced should be considered.

## Supporting Information

S1 Checklist STROBE Checklist. (DOCX)
