# Supplementary material for: Serological and Virological Evidence of Crimean-Congo Haemorrhagic Fever Virus Circulation in the Human Population of Borno State, Northeastern Nigeria
Source: PLoS Negl Trop Dis. 2016 Dec 7;10(12):e0005126. doi: 10.1371/journal.pntd.0005126 (PMC5142770; doi:10.1371/journal.pntd.0005126)
Supplement: S1 Checklist — (DOCX) [file pntd.0005126.s001.docx]

**Checklist S1:** STROBE Checklist

STROBE Statement—checklist of items that should be included in reports of observational studies

|  | Item No. | Recommendation | Manuscript  Section | Relevant text from manuscript |
| --- | --- | --- | --- | --- |
| **Title and abstract** | 1 | (*a*) Indicate the study’s design with a commonly used term in the title or the abstract | Title | Serological and virological evidence of Crimean-Congo Haemorrhagic Fever virus circulation in the human population of Borno state, Northeastern Nigeria |
|  |  | (*b*) Provide in the abstract an informative and balanced summary of what was done and what was found | Abstract | Despite several studies on the seroprevalence of antibodies against Crimean-Congo Haemorrhagic Fever virus (CCHFV) from humans and cattle in Nigeria, detailed investigation looking at IgG and IgM have not been reported. Additionally, there have been no confirmed cases of human CCHFV infection reported from Nigeria. Samples from sera (n=1189) collected from four Local Government Areas in Borno State (Askira/Uba, Damboa, Jere and Maiduguri) were assessed for the presence of IgG and IgM antibodies. The positivity rates for IgG and IgM were 10.6% and 3.5%, respectively. Additionally, sera from undiagnosed febrile patients (n=380) were assessed by RT-PCR assay for the presence of CCHFV RNA. One positive sample was characterised by further by next generation sequencing (NGS) resulting in complete S, M and L segment sequences. |
| Introduction | | | |  |
| Background/rationale | 2 | Explain the scientific background and rationale for the investigation being reported | Introduction | The first report of CCHFV in Nigeria occurred in 1970, when it was identified in various tick species, including Hyalomma spp. collected from market animals, and hedgehogs. Interestingly, very few cases of CCHF have been recorded in Africa; the majority are described from South Africa. The risk of CCHF in several African countries is poorly defined and infection with CCHFV is often undiagnosed or unreported in these regions. Importantly, CCHFV is a notorious cause of nosocomial infections especially when undiagnosed, and the virus presents a significant risk to health care workers. |
| Objectives | 3 | State specific objectives, including any prespecified hypotheses | Introduction | In the present study, seroprevalance was expanded across different Local Government Areas (LGAs) of Borno State and samples from patients with undiagnosed febrile illness were assessed for the presence of CCHFV RNA. |
| Methods | | | |  |
| Study design | 4 | Present key elements of study design early in the paper | Methods | Samples for serology testing were randomly selected from a cross section of humans in both rural and urban populations.  For molecular analysis, 380 serum samples from febrile patients in the acute phase of illness (fever and/or headache) were tested. |
| Setting | 5 | Describe the setting, locations, and relevant dates, including periods of recruitment, exposure, follow-up, and data collection | Methods | Samples were collected from 4 out of 27 LGAs within Borno State, namely:  Askira/Uba, Damboa, Jere, and Maiduguri  They presented to different clinics during 2010-2014 |
| Participants | 6 | (*a*) *Cohort study*—Give the eligibility criteria, and the sources and methods of selection of participants. Describe methods of follow-up  *Case-control study*—Give the eligibility criteria, and the sources and methods of case ascertainment and control selection. Give the rationale for the choice of cases and controls  *Cross-sectional study*—Give the eligibility criteria, and the sources and methods of selection of participants | Methods | Samples for serology testing were randomly selected from a cross section of humans in both rural and urban populations. |
|  |  | (*b*) *Cohort study*—For matched studies, give matching criteria and number of exposed and unexposed  *Case-control study*—For matched studies, give matching criteria and the number of controls per case |  |  |
| Variables | 7 | Clearly define all outcomes, exposures, predictors, potential confounders, and effect modifiers. Give diagnostic criteria, if applicable | Methods | Patients generally presented with febrile illnesses, and were screened for common aetiological agents, such as malaria and typhoid. |
| Data sources/ measurement | 8* | For each variable of interest, give sources of data and details of methods of assessment (measurement). Describe comparability of assessment methods if there is more than one group | Methods | **Serology:** Initial serology testing was conducted in Nigeria using an in-house ELISA to detect IgG and IgM antibodies against recombinant CCHFV nucleoprotein. A selection of samples was shipped to the UK for confirmatory analysis using both the in-house ELISA and a commercially-available assay system  **Virology:** Extraction of RNA was performed using the MagnaPure 96 small volume RNA kit . Target amplification was performed using primers to the CCHFV S segment |
| Bias | 9 | Describe any efforts to address potential sources of bias | - | Samples were blinded, so were identified only by a code during ELISA and PCR analysis. |
| Study size | 10 | Explain how the study size was arrived at | - | Study size was limited to the number of patients presenting at the relevant clinics and being negative for malaria and typhoid. |

Continued on next page

| Quantitative variables | | | 11 | | | Explain how quantitative variables were handled in the analyses. If applicable, describe which groupings were chosen and why | - | | N/A - for the analysis, qualitative readouts of data were used and groupings weren’t used. | |  |
| --- | --- | --- | --- | --- | --- | --- | --- | --- | --- | --- | --- |
| Statistical methods | | | 12 | | | (*a*) Describe all statistical methods, including those used to control for confounding | - | | The information within the manuscript did not require any specific statistical methods to be applied. | |  |
|  |  |  |  |  |  | (*b*) Describe any methods used to examine subgroups and interactions | - | |  |  |  |
|  |  |  |  |  |  | (*c*) Explain how missing data were addressed | - | |  |  |  |
|  |  |  |  |  |  | (*d*) *Cohort study*—If applicable, explain how loss to follow-up was addressed  *Case-control study*—If applicable, explain how matching of cases and controls was addressed  *Cross-sectional study*—If applicable, describe analytical methods taking account of sampling strategy | - | |  |  |  |
|  |  |  |  |  |  | (*e*) Describe any sensitivity analyses | - | |  |  |  |
| Results | | | | | | | | | | |  |
| Participants | | 13* | | | (a) Report numbers of individuals at each stage of study—eg numbers potentially eligible, examined for eligibility, confirmed eligible, included in the study, completing follow-up, and analysed | | Results | | 1,189 sera for CCHFV IgG and IgM.  380 samples assessed for the presence of CCHF viral RNA by RT-PCR. | |  |
|  |  |  |  |  | (b) Give reasons for non-participation at each stage | | - | | N/A | |  |
|  |  |  |  |  | (c) Consider use of a flow diagram | | - | | N/A | |  |
| Descriptive data | | 14* | | | (a) Give characteristics of study participants (eg demographic, clinical, social) and information on exposures and potential confounders | | Methods | | See Item 5. | |  |
|  |  |  |  |  | (b) Indicate number of participants with missing data for each variable of interest | | - | | N/A | |  |
|  |  |  |  |  | © *Cohort study*—Summarise follow-up time (eg, average and total amount) | | - | | N/A | |  |
| Outcome data | | 15* | | | *Cohort study*—Report numbers of outcome events or summary measures over time | | *-* | | N/A | |  |
|  |  |  |  |  | *Case-control study—*Report numbers in each exposure category, or summary measures of exposure | | *-* | | N/A | |  |
|  |  |  |  |  | *Cross-sectional study—*Report numbers of outcome events or summary measures | | Results | | Of the 1,189 sera from the 4 LGAs tested, 126 were positive for CCHFV IgG giving an overall seroprevalence of 10.6%, while 42 (3.5%) were seropositive for IgM.  Of 380 samples assessed for the presence of CCHF viral RNA by RT-PCR, a single sample (ID: N428) was positive. | |  |
| Main results | | 16 | | | (*a*) Give unadjusted estimates and, if applicable, confounder-adjusted estimates and their precision (eg, 95% confidence interval). Make clear which confounders were adjusted for and why they were included | | - | | N/A | |  |
|  |  |  |  |  | (*b*) Report category boundaries when continuous variables were categorized | | - | | N/A | |  |
|  |  |  |  |  | (*c*) If relevant, consider translating estimates of relative risk into absolute risk for a meaningful time period | | - | | N/A | |  |
| Other analyses | | 17 | | Report other analyses done—eg analyses of subgroups and interactions, and sensitivity analyses | | | | Results | | The genome assembly included three segments; the L Segment, M Segment and S Segment. Phylogeny was inferred using the maximum likelihood method based on the Tamura-Nei model and confidence assessed with the Bootstrap Test with 1000 resamplings. | |
| Discussion | | | | | | | | | | |  |
| Key results | 18 | | | Summarise key results with reference to study objectives | | | | Discussion | | Our serological results demonstrate the circulation of CCHFV in different LGAs of Borno State, Nigeria. The average positivity rates were 10.6% for IgG responses and 3.5% for IgM responses.  For molecular testing, sera samples from undiagnosed febrile patients were assessed for the presence of CCHFV using RT-PCR. Of the 380 samples tested, only a single sample showed a positive signal. |  |
| Limitations | 19 | | | Discuss limitations of the study, taking into account sources of potential bias or imprecision. Discuss both direction and magnitude of any potential bias | | | | Discussion | | Our report was conducted on samples collected from 4 LGAs in Nigeria. |  |
| Interpretation | 20 | | | Give a cautious overall interpretation of results considering objectives, limitations, multiplicity of analyses, results from similar studies, and other relevant evidence | | | | Discussion | | The similarity in antibody responses between our study and that conducted in 1974 indicates that despite the 38 year time period, similar frequencies of human CCHFV infection remain in Nigeria. This is consistent with recent evidence consensus of a moderate level of CCHFV in Nigeria. Although the data are focused on Borno State, we speculate that CCHFV is circulating in neighbouring countries which share common borders (Cameroon, Chad and Niger). |  |
| Generalisability | 21 | | | Discuss the generalisability (external validity) of the study results | | | | Discussion | | Whilst there has been strong serological evidence for CCHFV circulation in humans and cattle in Nigeria in the past, our data are the first to detect and directly sequence viral RNA in a human sample. Thus, this is the first report of human CCHFV infection in Nigeria. Importantly our work highlights that CCHFV should be considered as a potential cause of febrile illness in patients within the region and that further studies on the risk of CCHFV infection and how such risks could be reduced should be considered. |  |
| Other information | | | |  | | | | | | |  |
| Funding | 22 | | | Give the source of funding and the role of the funders for the present study and, if applicable, for the original study on which the present article is based | | | | - | | This study did not receive any specific funding. |  |

*Give information separately for cases and controls in case-control studies and, if applicable, for exposed and unexposed groups in cohort and cross-sectional studies.

**Note:** An Explanation and Elaboration article discusses each checklist item and gives methodological background and published examples of transparent reporting. The STROBE checklist is best used in conjunction with this article (freely available on the Web sites of PLoS Medicine at http://www.plosmedicine.org/, Annals of Internal Medicine at http://www.annals.org/, and Epidemiology at http://www.epidem.com/). Information on the STROBE Initiative is available at www.strobe-statement.org.
